# A Pharmacist-Led Medication Coaching Program Serving Senior Residents of Low-Income Housing

**DOI:** 10.1093/geroni/igaa057.2748

**Published:** 2020-12-16

**Authors:** Jennifer Bacci, Joshua Akers, Katie Mahan, Geoffrey Meer, Jeffrey Kinter, Anna Shields

**Affiliations:** 1 University of Washington, Seattle, Washington, United States; 2 Premera, Mountlake Terrace, Washington, United States; 3 Kelley-Ross Pharmacy, Seattle, Washington, United States; 4 Kelley-Ross Pharmacy Group, Seattle, Washington, United States; 5 Kaiser Permanente, Olympia, Washington, United States; 6 Kaiser Permanente Washington, Seattle, Washington, United States

## Abstract

In 2015, one independent community pharmacy partnered with the local Area Agency on Aging to provide medication coaching to low-income, culturally diverse, older adults living in 6 affordable housing buildings in the Seattle area. A pilot was conducted during the 2015-2016 fiscal year to determine the need for and feasibility of the service. Process outcomes, including patient and service demographics, medication-related problems, and pharmacist interventions, were captured via the pharmacists’ patient care documentation. Pharmacists had 34 total visits with 17 unique residents who were taking an average of 8.1 medications. Pharmacists identified 97 medication-related problems, averaging 5.7 problems per resident, and performed 88 interventions, averaging 5.2 interventions per resident. The findings of this pilot demonstrated the needs and feasibility of implementing pharmacists’ services within a housing organization structure and has resulted in the continuation and growth of the program.

